# GBM-Dx SIGNAL: Blood transcriptomics complementing neuroimaging to differentiate glioblastoma recurrence from treatment effects

**DOI:** 10.1016/j.gendis.2026.102029

**Published:** 2026-01-06

**Authors:** Dan Qi, James Murchison, Ekokobe Fonkem, Jason H. Huang, Erxi Wu

**Affiliations:** a Department of Neurosurgery and Neuroscience Institute, Baylor Scott & White Health, Temple, TX 76508, USA; b Department of Neurosurgery, Baylor College of Medicine, Temple, TX 76508, USA; c Department of Diagnostic Radiology, Baylor Scott & White Medical Center, Temple, TX 76508, USA; d Department of Neurology and Banner MD Anderson Cancer Center, University of Arizona, Phoenix, AZ 85006, USA; e College of Medicine and College of Pharmacy, Texas A&M University, College Station, TX 77843, USA; f Livestrong Cancer Institutes and Department of Internal Medicine, Dell Medical School, The University of Texas at Austin, Austin, TX 78712, USA

Glioblastoma (GBM), the most aggressive primary brain tumor in adults, accounts for more than half of malignant central nervous system neoplasms. Despite maximal treatment combining surgery and chemoradiotherapy, prognosis remains poor, with a median survival of approximately 14.6 months and a five-year survival rate below 7%. A major clinical challenge lies in accurately distinguishing true tumor recurrence from post-treatment-related effects (PTREs), such as radiation necrosis, pseudoprogression, inflammation, and edema. Misclassification can delay timely interventions and compromise survival, treatment efficacy, and quality of life.^1^^,^^2^ To our knowledge, no previous comprehensive study has integrated blood-based transcriptomic profiling with neuroimaging to address this diagnostic and research gap. This innovation aligns with the U.S. National Institutes of Health (NIH) priorities (PAR-25-175) for integrating liquid biopsy assays and imaging for precision cancer management and responds to the FY24 GBMRP Stakeholders Meeting Summary and Gaps for GBM: “Non-invasive biomarkers, liquid biopsy and imaging strategies” reported by the U.S. Department of Defense (DoD).

Currently, contrast-enhanced magnetic resonance imaging (CE-MRI) is the standard modality for GBM surveillance. However, its diagnostic specificity is limited by overlapping imaging features between recurrence and PTREs, often leading to repeated imaging, invasive biopsies, or misinformed clinical decisions—resulting in over- or under-treatment and unnecessary patient distress. To overcome these limitations, we developed GBM-Dx SIGNAL, a circulating transcriptomic platform designed to complement MRI and improve both the accuracy and timeliness of detecting GBM progression.

A major hurdle in blood-based transcriptomic profiling is the high abundance of globin mRNA, which can obscure the detection of informative transcripts. We addressed this by employing our whole-blood globin reduction (WBGR) protocol,^3^ which significantly improves the sensitivity and resolution of gene expression profiling. Using this method, our previous study^4^^,^^5^ identified 487 differentially expressed genes, distinguishing GBM patients from healthy controls, and 322 differentially expressed genes, differentiating stable disease from post-treatment recurrence (Fig. 1A and B; Table S1). Principal component analysis revealed a clear separation between recurrent and stable disease states, and gene expression profiles correlated strongly with patient survival, supporting the prognostic value. These findings demonstrate that transcriptomic alterations in peripheral blood can serve as a minimally invasive surrogate for intracranial disease activity.

**Figure 1:**
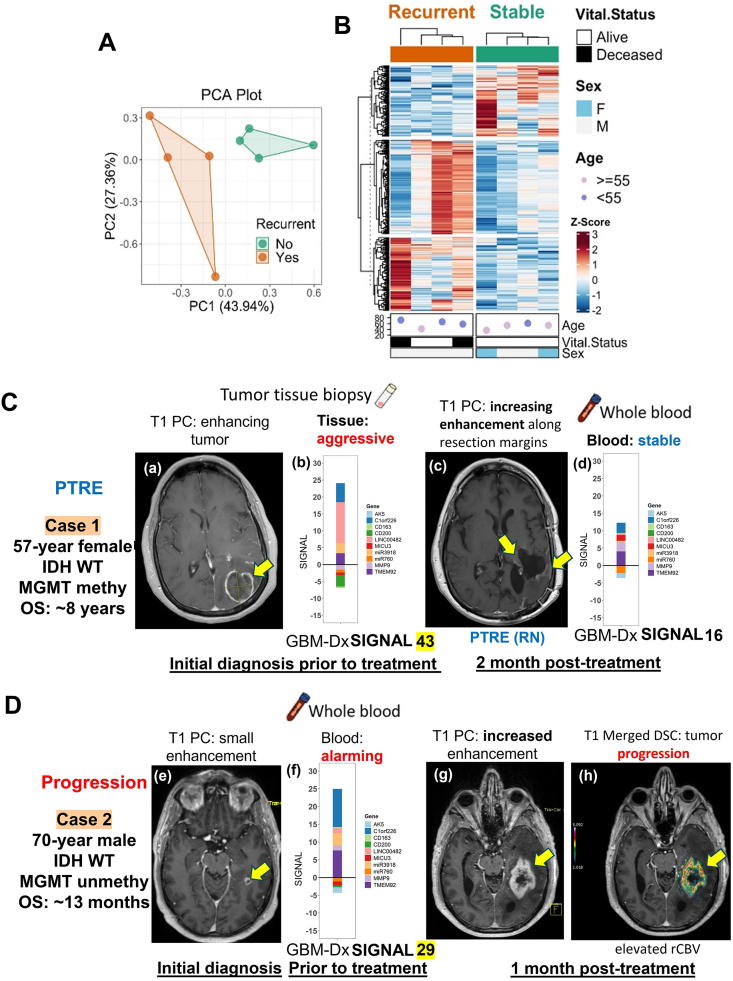
WBGR enhances detection of tumor recurrence in GBM blood, and signal in GBM analysis via liquid-biopsy (SIGNAL) scoring enables longitudinal disease monitoring aligned with neuroimaging findings. **(A)** Principal component analysis (PCA) of transcriptomic profiles differentiates recurrent GBM (rGBM) from treated, non-recurrent (stable) GBM cases. **(B)** Heatmap showing differentially expressed genes (*p* ≤ 0.05, fold change ≥ 2.0) distinguishing rGBM from treated stable cases. High expression is indicated in orange and low expression in blue. **(C, D)** Validation of GBM-Dx panel gene expression using independent blood and tumor tissue samples. Imaging scans were acquired at the indicated timepoints. Enhancing tumor lesions are bright areas and indicated with yellow arrows. Gene expression was validated using quantitative PCR. **(C)** Case 1: PTRE. A 57-year-old female with IDH wild-type, MGMT promoter-methylated GBM, and overall survival (OS) of ∼8 years. (a) Initial T1 post-contrast (T1 PC) MRI shows an enhancing lesion (yellow arrow), suggestive of a tumor. (b) Gene expression profiling from biopsy-confirmed tumor tissue shows a high GBM-Dx SIGNAL score of 43, indicating aggressive tumor biology. (c) At 2 months post-treatment, follow-up MRI reveals increased enhancement along the resection margins (yellow arrows), raising concerns for recurrence. (d) However, blood-based GBM-Dx profiling at the same timepoint yields a low SIGNAL score of 16, indicating a stable molecular profile, consistent with PTRE (radiation necrosis, RN) rather than active tumor. **(D)** Case 2: True tumor progression. A 70-year-old male with IDH wild-type, MGMT promoter-unmethylated GBM, and overall survival (OS) of ∼13 months. (e) Initial T1 PC MRI shows a small enhancing lesion (yellow arrow). (f) Blood-derived gene expression before treatment reveals an elevated SIGNAL score of 29, indicating alarming tumor activity despite modest imaging findings. (g) At one month post-treatment, increased enhancement is seen on T1 PC MRI (yellow arrow), and (h) merged dynamic susceptibility contrast (DSC)-MRI with elevated relative cerebral blood volume (rCBV) confirms tumor progression (red region), aligning with the earlier molecular signal from blood biomarkers. CBV color coding: blue/green = non-tumor; yellow = indeterminate; red = elevated CBV, indicating tumor. Contrast-enhancing lesions clinically confirmed as tumors and tumor tissues verified by pathology were described as “aggressive”, while lesions or tissues confirmed as treatment effects were described as “stable”. For blood samples, the study-defined SIGNAL threshold was applied: scores below the threshold were considered stable, and higher scores indicated aggressive or progressive molecular activity.

Building on our WBGR approach in GBM patient samples, we developed the GBM-Dx panel, as previously reported.^4^ This panel integrates established GBM-associated genes (*e.g.*, *MMP9*, *CD163*), novel candidates (*e.g.*, *AK5*, *MICU3*, *C1orf226*, *TMEM92*, *miR-3918*, LINC00482), and genes implicated in other cancers (*e.g.*, miR-760, CD200), thereby expanding the molecular landscape relevant to GBM biology. Using gene expression data from the cohort in our published study,^4^ we created the signal in GBM analysis via liquid-biopsy (SIGNAL) scoring system to quantify tumor presence and molecular activity with high sensitivity. In threshold assessment, scores ≤ 22 were consistent with patients with stable response (*n* = 6) (Table S2), whereas scores > 22 correlated with increased tumor aggressiveness (*n* = 4) (Mann–Whitney U test, *p* = 0.009375).

To demonstrate its clinical utility, we applied GBM-Dx SIGNAL in two representative GBM patients (Table S3) undergoing longitudinal post-treatment monitoring, illustrating how blood-based transcriptomic profiling can complement neuroimaging to differentiate PTRE from true tumor progression.

A 57-year-old female with IDH wild-type, MGMT promoter-methylated GBM had an overall survival of approximately 8 years. At the initial diagnosis (panel a in Fig. 1C), contrast-enhanced T1-weighted MRI (T1 PC) revealed a prominent enhancing lesion in the right frontal lobe (yellow arrow). Transcriptomic profiling from resected tumor tissue exhibited high expression of the GBM-Dx panel gene expression, corresponding to a SIGNAL score of 43 (panel b in Fig. 1C), consistent with aggressive tumor biology.

At two months post-treatment (panel c in Fig. 1C), follow-up T1 PC MRI demonstrated new enhancing foci along the surgical margins (yellow arrows), raising concern for recurrence. However, blood-based gene expression profiling at the same timepoint yielded a markedly lower SIGNAL score of 16 (panel d in Fig. 1C), indicating a stable molecular profile. The discordance between radiographic enhancement and molecular signal suggested that the MRI changes were due to PTRE, specifically radiation necrosis, rather than true tumor recurrence. This interpretation was later confirmed through longitudinal clinical follow-up and stable imaging findings, demonstrating the ability of GBM-Dx SIGNAL to provide critical discriminatory insight and help avoid unnecessary interventions.

A 70-year-old male with IDH wild-type, MGMT promoter–unmethylated GBM had a total overall survival of approximately 13 months. At baseline diagnosis (panel e in Fig. 1D), T1 PC MRI revealed a relatively small enhancing lesion (yellow arrow). Despite the modest radiographic appearance, blood-based molecular profiling revealed a SIGNAL score of 29 (panel f in Fig. 1D), indicating biologically aggressive tumor activity and early recurrence potential.

At one-month post-treatment, follow-up T1 PC MRI (panel g in Fig. 1D) showed marked lesion enlargement. Merged dynamic susceptibility contrast MRI with normalized relative cerebral blood volume mapping (panel h in Fig. 1D) demonstrated elevated relative cerebral blood volume in the enhancing region (red zone), strongly supporting a diagnosis of tumor progression rather than PTRE. These findings confirmed the earlier molecular warning provided by the elevated SIGNAL score.

These illustrative cases demonstrate the promise of GBM-Dx SIGNAL as a sensitive, non-invasive biomarker capable of reliably distinguishing GBM recurrence from PTRE, even when MRI findings are ambiguous. Acting as a longitudinal blood-based “molecular sentinel”, SIGNAL enables continuous monitoring of treatment response and recurrence risk, offering insights that imaging alone cannot provide.

Our ongoing efforts focus on embedding GBM-Dx SIGNAL into clinical workflows and potentially harmonizing it with RANO criteria. By reducing diagnostic uncertainty beyond MRI, GBM-Dx SIGNAL addresses a critical unmet need to distinguish progressive disease from treatment effects to enable timely interventions. Limitations include a modest sample size, requiring larger prospective studies to ensure reproducibility and generalizability. This study represents an early translational proof-of-concept, demonstrating a complementary relationship between blood transcriptomics and MRI rather than a fully integrative model. Future work will pursue quantitative correlations and multimodal modeling with expanded datasets while mitigating batch effects and gene-level variation. The platform is scalable and upgradeable, with transcriptomic panel expansion to enhance sensitivity and integrate AI models, enabling broad applicability. By bridging circulating transcriptomics with neuroimaging, GBM-Dx SIGNAL aligns with NIH and DoD priorities and establishes a framework for multimodal precision diagnostics in brain tumors.

## CRediT authorship contribution statement

**Dan Qi:** Writing – review & editing, Writing – original draft, Visualization, Software, Methodology, Investigation, Funding acquisition, Formal analysis, Data curation, Conceptualization. **James Murchison:** Software, Methodology, Investigation, Formal analysis. **Ekokobe Fonkem:** Resources, Investigation. **Jason H. Huang:** Supervision, Resources. **Erxi Wu:** Writing – review & editing, Supervision, Resources, Funding acquisition, Conceptualization.

## Ethics declaration

All human subject research was conducted in accordance with the guidelines approved by the Institutional Review Board (Approval No. 160016) at Baylor Scott & White Health (BSWH). Informed consent was obtained from all participants, and all procedures adhered to the principles of the Helsinki Declaration.

## Data availability

The de-identified data supporting the findings of this study are available from the corresponding authors upon reasonable request and through our previous publication^4^ under controlled access to ensure patient confidentiality.

## Funding

This work is supported by the BSWH Foundation (USA), the Corbett Estate Fund for Cancer Research (USA) (No. 62285-531021-41800, 62285-531021-61800 to E. Wu), and in part by the Cancer Prevention & Research Institute of Texas (CPRIT) (USA) (No. RP240537 to E. Wu).

## Conflict of interests

International patent WO/2022/140779 (https://patentscope.wipo.int/search/en/WO2022140779) covers methods for biomarker discovery; inventors E.W., D.Q., E.F., J.H.H. Erxi Wu is the member of *Genes & Diseases* Editorial Board. To minimize bias, he was excluded from all editorial decision-making related to the acceptance of this article for publication. The remaining authors declare no conflict of interest.
