# Supplementary material for: GBM-Dx SIGNAL: Blood transcriptomics complementing neuroimaging to differentiate glioblastoma recurrence from treatment effects
Source: Genes Dis. 2026 Jan 6;13(5):102029. doi: 10.1016/j.gendis.2026.102029 (PMC13137017; doi:10.1016/j.gendis.2026.102029)
Supplement: Multimedia component 1 [file mmc1.docx]

**Supplementary Material**

**Methods**

**Patient Cohort, GBM-Dx Scoring System, and Data Analysis**

Data used in this study were summarized in **Tables S1-3**. Differential gene expression analysis of previously published patient RNA-sequencing data was conducted using Student’s t-test, and results were visualized in the heatmap. Demographic and clinical information for the included patients is provided in **Table S1**.

In brief, for RNA-seq data, normalized expression values (counts per million, CPM) were log₂-transformed following between-sample quantile normalization using the DESeq2, limma, and edgeR packages. Fold change for each gene was calculated relative to the mean expression of the control group in our published work.^1^ The heatmap presented here represents a focused reanalysis of the existing RNA-seq dataset using the same R packages, restricted to the patient group, to illustrate differential expression between treated recurrent and treated stable-response cases. No additional RNA sequencing was performed in this study.

The GBM-Dx panel, developed from our prior publication, was analyzed by quantitative real-time PCR, with most samples processed together and one blood sample analyzed per patient in both previous^1^ and current studies. The PCR was performed as we previously described.^1^ Each RNA sample was analyzed in triplicate for each target gene, and the mean value of Ct was used for relative gene expression analysis using the$2^{-\Delta\Delta Ct}$ method.^2^ The $Ct$value of each target gene was normalized to an appropriate reference gene (GAPDH for mRNA, 18S rRNA for long non-coding RNA, and U6 for miRNA).^1^ The resulting $\Delta Ct$values were further normalized to the mean $\Delta Ct$of the control group to obtain $-\Delta\Delta Ct$. The$-\Delta\Delta Ct$ value^2^ corresponds to the log₂(fold change) for a given gene. The SIGNAL score for each sample was calculated as the sum of the absolute $-\Delta\Delta Ct$ values across the selected GBM-Dx genes.

The overall SIGNAL score can be calculated using a generalized scoring framework that integrates gene-level fold change, variability, and batch correction to produce a composite expression score for each sample. The formula is defined as:

$$SIGNAL\left( s \right)=\sum_{i=1}^{p} w_{i}\frac{max\left( 0,\left| x_{s,i}-\delta_{batch\left( s \right),c\left( i \right)} \right|-\tau_{i} \right)}{\sigma_{i}}$$

. Here:

- $SIGNAL(s)$ represents the overall composite score for sample $s$, capturing the total transcriptional deviation across all genes in the panel.
- $p$ denotes the total number of genes included in the scoring panel.
- $x_{s,i}$ is the log₂ fold change of gene $i$ in sample $s$, derived from $-\Delta\Delta Ct$ values to quantify relative expression changes.
- $w_{i}$ is the weighting factor for gene i, defining its relative contribution to the total score and set to 1 in this study.
- $\delta_{batch\left( s \right),c\left( i \right)}$ is the batch correction term that compensates for systematic differences across qPCR batches, where $c\left( i \right)$ indicates the gene class such as mRNA, lncRNA, or miRNA.
- $\tau_{i}$ represents the stability threshold for gene $i$, specifying the minimum expression change considered biologically meaningful and filtering out minor fluctuations as technical noise.
- $\sigma_{i}$ is the scaling factor, typically derived from the median absolute deviation (MAD) or standard deviation of control samples, used to normalize gene-specific variability.
- Max(0,·) is the noise-filtering function that ensures only expression changes exceeding the threshold​ $\tau_{i}$ contribute positively to the total SIGNAL score.

In the present dataset, a simplified version of the formula was applied $\left( w_{i}=1, \delta=0,\tau_{i}=0,\sigma_{i}=1 \right)$, yielding

$$SIGNAL(s)=\sum_{i=1}^{p} |x_{s,i}|$$

. This simplified form provides an interpretable composite score that captures the overall transcriptional activity across the selected gene panel in the currently examined small series of samples. In future analyses involving larger cohorts, the framework can incorporate MAD-based scaling, learned gene weighting, and batch correction to enhance robustness and enable consistent cross-study comparisons. The absolute value of the log₂-transformed expression ensures that both upregulated and downregulated genes contribute equally to the overall score, capturing the magnitude of dysregulation in either direction. This approach provides a robust, quantitative summary of transcriptomic activity that reflects tumor-related molecular changes in circulating blood.

SIGNAL scores were evaluated using two complementary analytical methods, based on patient data summarized in **Table S2**. First, a two-sided Mann–Whitney U test was applied to compare SIGNAL scores between patient subgroups and healthy controls. The mean SIGNAL score in the glioblastoma (GBM) patient group was 21.9 (rounded to 22), and this threshold yielded a statistically significant separation between stable and aggressive disease states (p = 0.009375). Second, receiver operating characteristic (ROC) analysis was performed using Youden’s J statistic to identify optimal classification thresholds. Despite the limited sample size, the threshold distinguishing healthy controls from GBM patients was calculated as 15.5 (rounded to 16), while the threshold separating stable from aggressive disease was 21.5 (rounded to 22).

Principal component analysis (PCA), heatmaps, and stacked bar plots were generated using R version 4.4.1 with standard statistical and visualization packages, e.g., ComplexHeatmap, DESeq2, ggpubr, rstatix, ggplot2 and pROC.^3-8^

**MRI Acquisition**

We retrospectively analyzed MRI scans acquired during routine clinical care, as detailed in our previously published study.^1^ Patient information related to these scans is summarized in **Table S3**.

All surveillance MRI scans were performed at Baylor Scott & White Health (BSWH) in accordance with the Brain Tumor Imaging Protocol (BTIP) recommendations.^9,10^ These protocols included acquisition of T1-weighted sequences (pre- and post-contrast), fluid-attenuated inversion recovery (FLAIR), diffusion-weighted imaging (DWI), and axial T2-weighted images.

Dynamic susceptibility contrast MRI (DSC-MRI) was performed with a single intravenous bolus of gadolinium-based contrast agent, following national consensus imaging recommendations. Acquisition parameters included: repetition time (TR) = 1.5 seconds, echo time (TE) = 30 ms, and flip angle = 30°. Relative cerebral blood volume (rCBV) maps were generated from T2*-weighted signal intensity-time curves.

To minimize inter-patient and inter-operator variability, rCBV values were normalized by dividing tumor rCBV by the mean rCBV of contralateral normal-appearing white matter (NAWM) in the centrum semiovale. This method produced normalized rCBV (nrCBV) maps with significantly reduced variability, enhancing reproducibility across patients. The nrCBV overlays were co-registered with structural T1-weighted MRI scans. Elevated nrCBV values were interpreted as indicators of increased vascularity and biologically active tumor regions.

Due to patient confidentiality, raw transcriptomic and imaging data are available upon request under controlled access with IRB approval.

**Table S1. Clinical Data Table-Blood Transcriptomics Heatmap**

| **ID** | **Diagnosis** | **Age^a^** | **Race** | **Gender** | **Recurrent or Stable** | **Treatment^b^** | **Vital Status^c^** | **Analysis^d^** |
| --- | --- | --- | --- | --- | --- | --- | --- | --- |
| ca002 | GBM | 66 | White | Male | Recurrent | Yes | Deceased | RNA-seq |
| ca003 | GBM | 58 | White | Male | Recurrent | Yes | Deceased | RNA-seq |
| ca004 | GBM | 42 | White | Male | Recurrent | Yes | Alive | RNA-seq |
| ca009 | GBM | 72 | White | Male | Recurrent | Yes | Deceased | RNA-seq |
| Mean age of Recurrent group |  | 59.5 |  |  |  |  |  |  |
| Sex distribution (F:M) |  |  |  | F0: M4 |  |  |  |  |
| ca005 | GBM | 54 | White | Male | Stable | Yes | Alive | RNA-seq |
| ca007 | GBM | 37 | White | Female | Stable | Yes | Alive | RNA-seq |
| ca010 | GBM | 61 | White | Male | Stable | Yes | Alive | RNA-seq |
| ca011 | GBM | 54 | White | Female | Stable | Yes | Alive | RNA-seq |
| Mean age of stable group |  | 51.5 |  |  |  |  |  |  |
| Sex distribution (F:M) |  |  |  | F2: M2 |  |  |  |  |

a, age at lab draw (years); b, treatment received or not before taking blood samples; c, as of the data analysis date; d, sequencing data from our previous publication^1^; F, Female; M, male; NA, not available or not applicable.

**Table S2. Clinical Data Table-Blood Signal Computation**

| **ID** | **Diagnosis** | **Age^a^** | **Race** | **Gender** | **Recurrent** | **Treatment^b^** | **Vital Status ^c^** | **GBM-SIGNAL** | **Analysis^d^** |
| --- | --- | --- | --- | --- | --- | --- | --- | --- | --- |
| ct004 | Control | 66 | White | Female | NA | NA | NA | 7 | RT-qPCR |
| ct008 | Control | 30 | White | Male | NA | NA | NA | 5 | RT-qPCR |
| ct010 | Control | 69 | White | Female | NA | NA | NA | 5 | RT-qPCR |
| ct016 | Control | 54 | White | Male | NA | NA | NA | 4 | RT-qPCR |
| ct005 | Control | 47 | White | Female | NA | NA | NA | 15 | RT-qPCR |
| ct013 | Control | 58 | White | Male | NA | NA | NA | 12 | RT-qPCR |
| Mean age of control group |  | 54 |  |  |  |  |  |  |  |
| Sex distribution (F:M) |  |  |  | F3: M3 |  |  |  |  |  |
| ca001 | GBM | 72 | White | Female | No | No | Deceased | 28 | RT-qPCR |
| ca006 | GBM | 57 | White | Male | Yes | No | Deceased | 23 | RT-qPCR |
| ca007 | GBM | 37 | White | Female | No | Yes | Alive | 19 | RT-qPCR |
| ca011 | GBM | 54 | White | Female | No | Yes | Alive | 22 | RT-qPCR |
| ca008 | GBM | 57 | White | Female | Yes | Yes | Alive | 16 | RT-qPCR |
| ca013 | GBM | 59 | White | Female | No | Yes | Alive | 21 | RT-qPCR |
| ca014 | GBM | 70 | White | Female | No | Yes | Deceased | 19 | RT-qPCR |
| ca015 | GBM | 37 | African American (Black) | Male | Yes | Yes | Alive | 23 | RT-qPCR |
| ca016 | GBM | 20 | White | Male | No | Yes | Alive | 19 | RT-qPCR |
| ca018 | GBM | 70 | White | Male | No | No | Deceased | 29 | RT-qPCR |
| Mean age of GBM group |  | 53.3 |  |  |  |  |  |  |  |
| Sex distribution (F:M) |  |  |  | F6: M4 |  |  |  |  |  |

a, age at lab draw (years); b, treatment received or not before taking blood samples; c, as of the data analysis date; d, PCR primers same to our previous publication^1^; F, Female; M, male; NA, not available or not applicable.

**Table S3. Clinical Data Table-Blood and Imaging Comparison Analysis.**

| **ID** | **Diagnosis** | **Age^a^** | **Race** | **Gender** | **Analysis^d^** |
| --- | --- | --- | --- | --- | --- |
| ca008 | GBM | 57 | White | Female | RT-qPCR, imaging |
| ca018 | GBM | 70 | White | Male | RT-qPCR, imaging |

a, age at lab draw (years); d, PCR primers same to our previous publication^1^.

**References:**

1. Qi, D. *et al.* Transcriptomic analyses of patient peripheral blood with hemoglobin depletion reveal glioblastoma biomarkers. *npj Genomic Medicine* **8**, 2 (2023).

2. Livak, K.J. & Schmittgen, T.D. Analysis of relative gene expression data using real-time quantitative PCR and the 2(-Delta Delta C(T)) Method. *Methods* **25**, 402-8 (2001).

3. Love, M.I., Huber, W. & Anders, S. Moderated estimation of fold change and dispersion for RNA-seq data with DESeq2. *Genome Biol* **15**, 550 (2014).

4. Gu, Z., Eils, R. & Schlesner, M. Complex heatmaps reveal patterns and correlations in multidimensional genomic data. *Bioinformatics* **32**, 2847-9 (2016).

5. Kassambara, A. ggpubr: 'ggplot2' Based Publication Ready Plots. (2020).

6. Kassambara, A. rstatix: Pipe-Friendly Framework for Basic Statistical Tests. (2021).

7. Wickham, H. *ggplot2: Elegant Graphics for Data Analysis*, (Springer-Verlag New York, 2016).

8. Robin, X. *et al.* pROC: an open-source package for R and S+ to analyze and compare ROC curves. *BMC Bioinformatics* **12**, 77 (2011).

9. Ellingson, B.M. *et al.* Consensus recommendations for a standardized Brain Tumor Imaging Protocol in clinical trials. *Neuro Oncol* **17**, 1188-98 (2015).

10. Kaufmann, T.J. *et al.* Consensus recommendations for a standardized brain tumor imaging protocol for clinical trials in brain metastases. *Neuro Oncol* **22**, 757-772 (2020).
